# Reducing the strain on calorie restriction

**DOI:** 10.1093/lifemeta/loag006

**Published:** 2026-03-18

**Authors:** John R Speakman, Sharon E Mitchell

**Affiliations:** School of Biological Sciences, University of Aberdeen, Aberdeen AB24 2TZ, United Kingdom; Shenzhen Key Laboratory of Metabolic Health, Center for Energy Metabolism and Reproduction, Shenzhen Institutes of Advanced Technology, Chinese Academy of Sciences, Shenzhen, Guangdong 518055, China; State Key Laboratory of Molecular Developmental Biology, Institute of Genetics and Developmental Biology, Chinese Academy of Sciences, Beijing 100101, China; Institute of Health Sciences, China Medical University, Shenyang, Liaoning 110122, China; School of Biological Sciences, University of Aberdeen, Aberdeen AB24 2TZ, United Kingdom

**Keywords:** calorie restriction, mouse, strain, sample size, artefact

## Abstract

Calorie restriction (CR) is a nutritional intervention known to delay aging and extend lifespan across a wide range of species, raising the possibility of similar benefits in humans. This apparent universality has been questioned by studies reporting shortened lifespan under CR in certain mouse strains. Here, we provide a short perspective on these conflicting findings. Using simple simulation analyses, we explored the apparent strain-specific effects on CR outcomes. Our results illustrate how experimental factors have confounded previous interpretations and led to overemphasis on genotype effects. Reproducible CR studies are crucial for understanding the true potential of CR as a broadly applicable intervention in aging.

Calorie restriction (CR), the reduction of calorie intake below baseline requirements while maintaining intake of micronutrients (sometimes also called dietary restriction), is the most robust environmental manipulation that impacts health span [1] and increases both median and maximal lifespan [2]. Discovered over 100 years ago in rodents [3], CR has been shown to extend the lifespan of a variety of mammals [2], including cows, dogs, and non-human primates [4, 5], leading to hopes that it may also be effective in humans. A similar manipulation called calorie dilution also extends the lifespan of many invertebrates. However, calorie dilution has different phenotypic impacts to CR [6] and may work by different mechanisms unrelated to CR [7]. Moreover, the impact of calorie dilution is not universally positive, with houseflies [8] and mice [9] showing no, or a negative, lifespan response to it.

In 2010, two landmark papers were published, which showed that the impact of CR in inbred mice was critically dependent on the strain of mouse used [10, 11]. These papers have been extremely influential in the aging field. The study by Liao *et al.* [10], for example, has been cited more than 400 times. This importance is because a key feature of these papers was the surprising result that for many strains the impact was negative—that is, CR shortened lifespan rather than lengthening it. These studies raised an important issue because if the impact of CR was heavily impacted by genetic background (= inbred strain), it would be likely that the impact would vary between individual humans. Some humans embarking on a program of CR might then ultimately be shortening their lifespan rather than extending it. On a brighter note, for researchers, the differential responses of the recombinant inbred strains of mice led to the exciting possibility that it would be ­possible to identify important genetic factors that underpin positive and negative responders—thereby allowing us to screen individuals to identify those who would benefit the most from this intervention, and perhaps more importantly, those who would have no benefit or a negative impact. Fifteen years after the landmark papers were published, that hope has not been realized. In this perspective, we discuss why, and in so doing cast doubt on the whole notion of large positive and negative strain effects of genetic background on the CR outcomes in mice.

The two landmark studies involved recombinant inbred strains of mice known as the ILSXISS strains. These strains were derived from a long-term selection experiment for alcohol tolerance [12]. Strains were bred for high (ILS) and low (ISS) tolerance and then crossed (ILS × ISS) to generate 72 inbred strains. Liao *et al.* [10] and Rikke *et al.* [11] studied the effect of CR on these strains. Liao *et al.* studied males and females of 41 strains [10], while Rikke *et al.* focused on only females of 42 strains [11]. Twenty-seven of the studied strains were common to both labs. They both observed that some strains had the classical extension of lifespan, but others showed no impact, and in yet others, lifespan was shortened. Surprisingly, given previous work suggesting that lifespan in mice was usually extended by CR, the majority of strains showed either no effect or a shortening of lifespan. A major issue, however, as we pointed out shortly after the papers were published, was that (i) the correlation between the effects of CR in the two sexes was unexpectedly weak, and (ii) there was no correlation at all between the responses of specific strains across the two laboratories [2]. For example, strain 110 lived 440 days longer under CR in the study by Liao *et al.* [10], but lived 200 days shorter under CR in that by Rikke *et al.* [11].

This lack of repeatability across labs is a major issue because it undermines any further work on the strain effect, because it is unclear whether any particular strain is a positive or negative responder. This, we suggest, has hindered any progress in using these strains. It is important then to ask where this lab-to-lab variation comes from. There has been much discussion about the exact protocol used in these studies, which involved the unusual (for the time) procedure of group housing the individuals [13, 14]. To that point, group housing was unusual because it is impossible to measure individual levels of restriction for group housed animals and it might lead to fighting over the restricted food levels, particularly among males [15]. On the other hand, group housing has become more common nowadays because it is suggested that mice housed individually may suffer social isolation stress. Indeed, several studies have shown that individually housed mice exhibit greater anxiety and depressive-like behaviour, accompanied by altered neuroplasticity-related gene expression [16]. Liao *et al.* [14] suggested that group housing was unlikely to be an issue in their studies for several reasons. First, if competition for food was an issue, one might expect that dominant individuals would obtain more food than subordinates, and this would lead to elevated variance in body weight among the restricted animals. However, the varia­tion in body weight in the restricted mice was not different from that of *ad libitum* (AL)-fed mice. Second, there was no difference in lifespan between the heaviest and the lightest individuals in any cage. Third, they did not observe overt fighting when the food was delivered. Finally, for four strains that showed lifespan shortening effect, they presented data for both single- and group-housed individuals, indicating lifespan shortening in three of the four strains under single-housing conditions. Nevertheless, Rikke *et al.* [11] noted that some animals had to be removed from their study due to fighting-related deaths, and both studies censored animals that died within 100 days of the CR procedure starting. In our CR studies, no deaths occurred in individually housed mice within 100 days of initiating the CR procedure, suggesting that there is something different between group- and single-housing conditions.

However, in addition to the group housing issue, a major overlooked problem with these studies (mentioned briefly by Mattson [13]) was the sample size of individuals involved for each strain. This was justified by the claim that the goal of the study was to establish genetic variation linked to CR, and in that context, it is better to study more strains, even at the expense of fewer mice per strain [14]. In the study by Liao *et al.* [10], the maximum number of mice used to establish the lifespan effect was only five AL-fed mice and five CR-fed mice. In many cases, the sample size under CR was smaller (due to censoring early deaths, perhaps owing to fighting), and for two strains, the “strain data” consisted of the lifespan of a single mouse! Could these small sample sizes explain the weak correlation between males and females and the poor inter-laboratory repeatability?

To explore these possibilities, we performed some modeling of the impact of sample size on the distribution of responses to CR, the correlation between males and females and the repeatability across labs. To simulate the effects, we pooled all the lifespan data from Liao *et al.* [10] for the AL-fed mice and all the data for the CR-fed mice for each sex separately. This created four approxi­mately normal distributions of lifespans (Fig. 1). The actual distributions showed considerable negative skew, and this appeared to be much greater in CR-exposed animals than in AL-fed animals. In CR-exposed animals, quite a lot of the animals lived less than 600 days, while such short lifespans were rarer in AL-fed mice.

**Figure 1:**
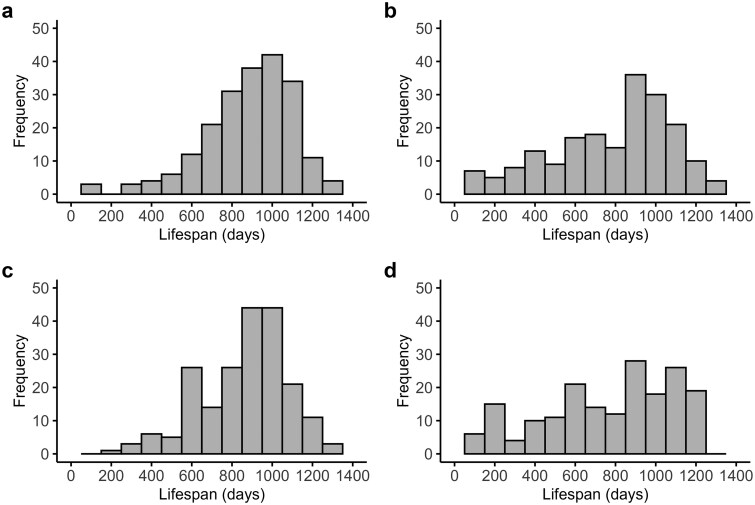
Pooled data for lifespan across all strains for both male and female mice exposed to AL and CR feeding. (a) AL-fed males. (b) CR-fed males. (c) AL-fed females. (d) CR-fed females. Data are from Liao *et al*. [10].

We characterized these distributions by their means and standard deviations, and then randomly selected five values for the AL-fed and five for the CR-fed mice of each sex from these distributions. This sampling process was repeated 41 times to generate data for males and females of 41 simulated “strains.” We then censored these data by reducing sample sizes to match the sample sizes in Liao *et al.* [10] for their actual strains and then calculated the mean lifespan of AL and CR samples of mice for each “strain.” This allowed us to calculate the extent of lifespan shortening or extension for each strain and sex (Fig. 2).

**Figure 2:**
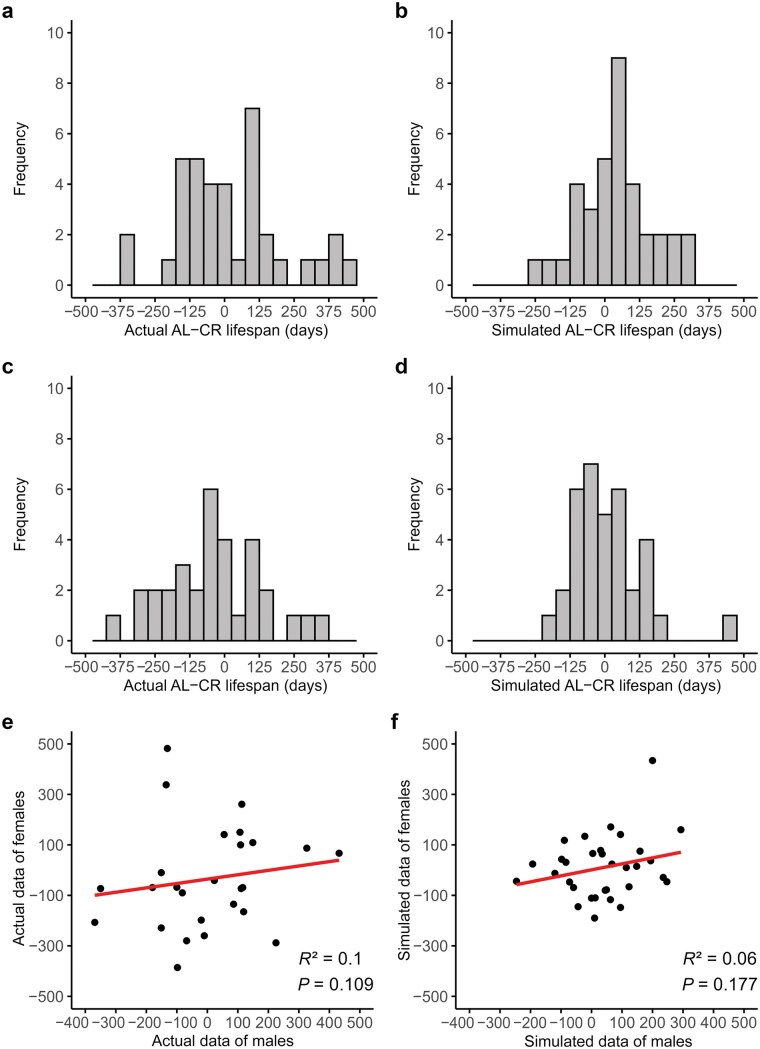
Distribution of lifespan responses (AL-fed mice minus the CR-fed mice) between the strains. Negative values, therefore, refer to lifespan-extending effects of CR, and positive values refer to lifespan shortening. (a) Actual data of males from Liao *et al*. [10] (*n *= 37). (b) Simulated data of males (*n *= 36). (c) Actual data of females (*n *= 34). (d) Simulated data of females (*n *= 35). (e and f) Correlations between males and females for the actual (*n *= 27, e) and simulated (*n *= 32, f) data.

First, we looked at the distribution of the differences in lifespan, that is, shortening or extending, between the actual strains (Fig. 2a and c) and the simulated strains (Fig. 2b and d) in males and females, respectively. The distributions of responses were very similar between the actual and simulated strains. The means of the actual and simulated distributions did not differ significantly (males: paired *t *= 0.45, *P *= 0.65; females, *t *= 0.38, *P *= 0.71) but there was a small significantly higher variance in the actual data (males: Levene’s *F*-test, *F *= 4.78, *P *= 0.03; females: *F *= 5.93, *P *= 0.018: males not significant with Bonferroni correction for multiple tests). Second, we compared the correlation of the lifespan effect of CR on males and females of the actual strains studied by Liao *et al.* [10] (Fig. 2e) versus the simulated strains (Fig. 2f). These plots were almost identical, and in a pooled general linear model, the source of the data (simulated versus actual) had no significant impact on the association between males and females (*F *= 0.1, *P *= 0.76).

The similarity of the actual data to the simulated data leads us to suggest that the strain effects detected in Liao *et al.* [10] are largely sampling artefacts. Effects attributed to “strain” are likely mostly due to random sampling and a very small sample size for each strain and sex. This would explain the poor correlation of male and female responses, why there was enormous variation between strains including life shortening, and crucially the lack of repeatability across the labs. The fact that the variance in the actual data was marginally higher suggests that there may be still a small strain effect, but it is negligible compared to the sampling artifact. This discrepancy in variance between the real and simulated data might also be explained by the fact that a simple normal distribution did not exactly reflect the distributions of lifespans pooled across all strains (Fig. 1). The possibility that the original “strain” data is an artifact would also explain why there has been zero progress in delineating the differences between responder and non-responder ILSXISS strains, despite valiant attempts to do so [17], because actually minimal strain effects exist in these data sets.

Since these seminal works, there have been other attempts to replicate the findings, including one using a larger sample size [18]. These authors took the four strains from Liao *et al.* [10] where CR resulted in the largest lifespan shortening effect, and repeated the experiment with sample sizes of ≥ 30 AL- and CR-fed mice per strain (but again grouped the animals five to a cage). Given that they studied both sexes of four strains, this involved eight strains by sex comparisons, where the expectation was eight out of eight lifespan reductions. They found that four of the eight comparisons showed the conventional life-extending impact of CR, three of eight showed no effect, and only one of eight replicated the lifespan shortening impact described by Liao *et al.* [10]. Even in this strain, the magnitude of the previous life reduction was strongly attenuated. Moreover, this could be an example where aggression due to the group housing protocol shortened lifespan of those under CR. Variations in the experimental parameters of the three studies [10, 11, 18] investigating the lifespan response of ILSXISS mice to 40% CR are summarized in Table 1.

**Table 1:** Experimental parameters of lifespan studies investigating response to 40% CR in the ILSXISS mice [10, 11, 18].

Sex	Number of strains	Sample size	Group housing	Diet	Age initiated (days)	Regime	AL–LS (days)	CR–LS (days)	Increased LS Number of strains, extent (%)	Decreased LS Number of Strains, extent (%)	Reference
M	41	Most 5 (range 2–9)	5/cage	HT7912, unfortified	60–150	No stepdown, fed daily DP, fixed 12 months	504–1152	217–1215	2/41, +53%	11/41, –43%	[10]
F	39						407–1208	113–1225	8/41, +38%	10/41, –60%	
F	42	AL, most 10 CR, most 12	AL, 5/cage CR, 6/cage	HT7012	28–40	3× week, daily 1 year, fixed 18 months	490–1020	380–1070	9/42, +41%	8/42, –36%	[11]
M	4^a^	30–45	5/cage	NIH-31, unfortified	42	No stepdown, fed daily DP, fixed 18 months	Median 882–1069	Median 936–1045	1/4, +9%	1/4, –22%	[18]
F	4^a^	30–45					Median 620–1060	Median 929–1079	3/4, +28%	0	

AL, *ad libitum*; CR, calorie restriction; LS, lifespan; HT, Harlan Teklad; DP, dark phase; 3× week regime = double ration Monday & Wednesday, triple Friday.

a Strains with the highest reduction in LS from Ref. [10].

What are the implications of the modeling presented here and the work of Unnikrishnan *et al.* [18]? Eleven years prior to the landmark studies of Liao *et al.* and Rikke *et al.*, there was a major study on the strain effects of CR [19]. They studied four mouse strains and three rat strains, and used more than 60,000 individually housed animals in their experiments. They showed that CR always had a positive effect on lifespan, but the impact in the inbred C57BL/6-derived strain was significantly greater than in the DBA/2 strain, with hybrid strains showing intermediate responses. If strain effects were known to exist all along, why did the studies by Liao *et al.* and Rikke *et al.* make such a big splash? The basic difference was that these two papers were the first to suggest that, in many cases, the effects of CR might be negative (as opposed to variable positive effects). Our modeling here, and the broad lack of replication of the negative impacts by Unnikrishnan *et al.* [18] suggest that these negative “strain effects” may be almost completely sampling artefacts, potentially combined by effects of group housing and feeding protocols on the responses of the animals to CR.

This analysis leads to several take-home messages. First, using inadequate sample sizes to characterize lifespan responses can lead to spurious effects. It is remarkable that these studies have had such a large impact in the field, despite the shaky foundation that they were built on. Second, group-housing mice under CR may blunt the impact on lifespan due to stress. It may now be time to explore if it is ethically acceptable to continue this protocol. Third, although CR does have some downsides [20] when appropriate sample sizes and protocols are used, CR seems to have almost universally positive effects on health and lifespan in mice. Summarizing 80 years of CR research, Speakman *et al.* found a consistent extension to lifespan in rodents, which increased with the degree of restriction [21]. An update of the last decade of CR research (Table 2) confirms variability in the lifespan response, but no significant shortening was reported. This may give some reassurance to people considering engaging in a CR protocol to enhance longevity. Studies vary considerably in protocol; in addition to sample size and group housing, age of initiation and feeding regime may affect the response (Table 2). The magnitude of variation in the positive impacts of CR between studies likely involves some impact of strain, but it is dominated by other protocol impacts.

**Table 2:** The effect of CR on lifespan in mice.

Year	Strain	CR level	Sex	%LS increased	CR initiated (days)	Regime	Sample size	Group housing	Diet	Reference
2016	C57BL/6J	20	F	40	180	10% decrease per week, fed daily at 7:30 a.m.	50	3–4/cage	HT 2018	[23]
		40	F	−4						
		20	M	24						
		40	M	19						
	DBA2/J	20	F	31	180	10% decrease per week, fed daily at 07:30	58	3–4/cage	HT 2018	
		40	F	26			61			
		20	M	13			59			
		40	M	16			61			
2019	*NQO1*-ko	40	M	14	210–280	Food monitored bi-weekly	30	2–4/cage	NIH-31	[24]
	Littermates	40	M	-			33			
2019	C57BL/6J	30	M	33	280	2-week stepdown, fed daily at 3:00 p.m., fixed 14 months	59	Single	NIA	[25]
		30	M	35			62		WIS	
2020	*Nrf2*-ko	30	M	16	210–280	10% decrease per week, fed daily at 06:30 − 08:30	40	3–4/cage	NIH-31	[26]
	WT	30	M	4			39	3–4/cage	NIH-31	
2021	C57BL/6J	30	M	20	120	10% decrease per week, fed daily at 07:00	NR	NR	HT 2018	[27]
2022	C57BL/6J	30, No fast (NF)	M	10	98	Automated feed system, NF = 300 mg/160 min, fed start day or night as appropriate	36	Single	BioServ F0075	[28]
		30, 12h Fast-Day		19						
		30, 22h Fast-Day		21						
		30, 12h Fast-Night		34						
		30, 22h Fast-Night		35						
2022	WT	25	M	20	60	Control fixed 16% decrease calories, fed 3× week at 7:00 a.m.	61	Single	AIN-93M	[29]
	*Sirt3* ^−/−^			29			75			
2023	C57BL/6J	20	F	4	427	Fed at 08:30	35	4/cage	NIH-31	[30]
		20 × 2 feeds		5		Fed at 8:30 a.m. and 4:30 p.m.	37			
2024	Diversity Outbred	20	F	18	180	Daily, Monday–Thursday Triple Friday	189	8/cage	LabDiet (5K0G)	[22]
		40		36			182			

Studies reported are from 2016 to date (update of literature [21]). Increase in lifespan is shown as median unless otherwise stated. CR, calorie restriction; LS, lifespan; *NQO1*-ko, NAD(P)H (nicotinamide adenine dinucleotide phosphate): quinone oxidoreductase 1 knockout; *Nrf2*, nuclear factor erythroid-2-related factor 2; *Sirt3*, Sirtuin3. HT 2018 = 2018 Teklad Global 18% Protein Rodent Diet, Harlan Teklad. NIA diet (7.6% energy from sucrose, 17.7% from fat, 3.4 kcal/g). WIS diet (46% energy from sucrose, 24.4% from fat, 3.9 kcal/g). The sample size shows the number of mice in the CR group. NR, not reported.

Studies of genetically diverse female mice have shown that there are substantial differences in the impacts of CR between individuals and that genetic background has a greater influence than CR on the attained lifespan [22]. A major problem with using these Diversity Outbred mice, however, is that you cannot kill the same mouse twice under both conditions. Hence, for a given individual (genotype), the lifespan-extending impact of CR rela­tive to how long it would have lived if fed AL is unclear. Using inbred strains overcomes this issue because the individuals within a given strain have the same genotype, allowing exposure of the same genotype to multiple dietary interventions. Hence, by using multiple strains that differ in the impact of CR can reveal the likely gene by exposure interaction, which would indicate the likely lifespan-extending mechanisms involved. Strain differences in the extent of the benefits of CR may give us a window into how CR works, but choosing the right strains to achieve this will be critical. Moreover, it is important to recognize the diversity of alternative CR protocols and that these may interact differently with genotype. Teasing apart the genetic impacts by comparing responses of different strains will be complicated by the wide diversity of different protocol effects. A starting point for moving the field forward might be for studies using CR to adopt a common protocol. We suggest that it would be best to involve single housing animals where the exact intake of each animal can be quantified, and there are no detrimental effects of inter-individual aggression. In addition, we suggest that mice should be fed a ration every day, avoiding protocols where mice may be without food completely for one or more days. Harmonizing the protocol would then allow an easier dissection of the importance of other biological impacts.
